# Brain-Oct-Pvt: A Physics-Guided Transformer with Radial Prior and Deformable Alignment for Neurovascular Segmentation

**DOI:** 10.3390/bioengineering13030332

**Published:** 2026-03-13

**Authors:** Quan Lan, Jianuo Huang, Chenxi Huang, Songyuan Song, Yuhao Shi, Zijun Zhao, Wenwen Wu, Hongbin Chen, Nan Liu

**Affiliations:** 1 Department of Neurology, Fujian Medical University Union Hospital, Fuzhou 350001, China; xmdylanquan@163.com (Q.L.); zhaozijun0719@163.com (Z.Z.); wenwenwu2026@163.com (W.W.); 2 Institute of Clinical Neurology, Fujian Medical University, Fuzhou 350122, China; 3 Clinical Research Center for Precision Diagnosis and Treatment of Neurological Diseases of Fujian Province, Fujian Medical University Union Hospital, Fuzhou 350001, China; 4 Department of Rehabilitation, Fujian Medical University Union Hospital, Fuzhou 350001, China; 5 Department of Neurology, The First Affiliated Hospital of Xiamen University, Xiamen 361003, China; arnohuangjianuo@gmail.com (J.H.); supermonkeyxi@xmu.edu.cn (C.H.); 30320212200061@stu.xmu.edu.cn (S.S.); syhhh@stu.xmu.edu.cn (Y.S.)

**Keywords:** neurovascular OCT, deformable convolution, polar processing, boundary attention, medical imaging

## Abstract

The primary objective of this study is to develop a specialized deep learning framework specifically adapted for the unique physical characteristics of neurovascular Optical Coherence Tomography (OCT) imaging. Although Polyp-PVT, originally designed for polyp segmentation, shows promise for OCT analysis, it faces limitations in neurovascular applications. The default RGB input wastes resources on duplicated grayscale data, while its fixed-scale fusion struggles with vascular curvature variations. Furthermore, the attention mechanism fails to capture radial vessel patterns, and geometric constraints limit thin boundary detection. To address these challenges, we propose Brain-OCT-PVT with key innovations: a single-channel input stem reducing parameters by two-thirds; a Radial Intensity Module (RIM) using polar transforms and angular convolution to model annular structures; and a Deformable Cross-scale Fusion Module (D-CFM) with learnable offsets. The Boundary-aware Attention Module (BAM) combines Laplace edge detection with Swin-Transformer for sub-pixel consistency. A specialized loss function combines Dice Similarity Coefficient (Dice), BoundaryIoU on 2-pixel dilated edges, and Focal Tversky to handle extreme class imbalance. Evaluation on 13 clinical cases achieves a Dice score of 95.06% and an 95% Hausdorff Distance (HD95) of 0.269 mm, demonstrating superior performance compared to existing approaches.

## 1. Introduction

**Figure 1:**
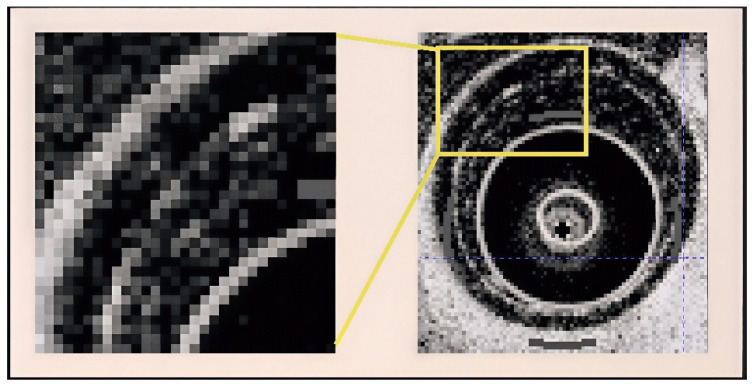
Characteristic trilaminar appearance of normal coronary arterial walls on OCT.

Optical Coherence Tomography (OCT) has emerged as a transformative modality for intracranial vascular imaging [1], offering distinct advantages over conventional angiography techniques. Its ability to visualize vessel wall microstructures at 10–15 μm resolution through near-infrared interferometry represents a significant leap forward in neurovascular diagnostics (Figure 1). The clinical potential of vascular OCT was first established through landmark coronary imaging studies, with recent cerebrovascular adaptations demonstrating unique capabilities including radiation-free operation, immunity to metal artifacts from intracranial implants, and real-time volumetric acquisition during catheter pullback [2].

The imaging physics of OCT introduce characteristic noise patterns that fundamentally differ from other modalities [3]. Speckle noise arises from the multiplicative interference of backscattered coherent light waves, creating a granular texture that obscures subtle anatomical boundaries. This phenomenon differs markedly from the additive Gaussian noise in Magnetic Resonance Imaging (MRI) [4] or Poisson-distributed noise in Computed Tomography (CT) systems. In cerebrovascular applications, speckle presents particular challenges due to the delicate nature of intracranial vessel walls, where the media layer may be as thin as 100–200 μm. The noise spectral characteristics follow a Rayleigh distribution with spatial correlations influenced by the coherence length of the laser source and scanning protocol parameters.

Current vascular segmentation methods face significant limitations when applied to neurovascular optical coherence tomography (OCT) analysis, despite their success in other medical imaging domains. Conventional PVT-based models like PVT-Cascade [5], while demonstrating strong performance for general medical image segmentation tasks, exhibit several critical shortcomings when processing OCT data. The fundamental issue stems from their architectural incompatibility with OCT’s unique physical characteristics. Most notably, these models employ standard RGB input processing, creating unnecessary computational overhead when handling OCT’s intrinsically single-channel grayscale data, where all three color channels contain identical information. More importantly, they lack specialized mechanisms to effectively capture OCT’s distinctive radial intensity patterns, particularly the consistent configuration of dark lumens surrounded by bright concentric wall gradients that characterize cerebrovascular anatomy.

Recent advanced segmentation models, exemplified by ADCFormer [6] (2025) and SAM-OCTA2 [7] (2024), while incorporating sophisticated transformer architectures, still exhibit critical deficiencies in Brain-OCT applications. These state-of-the-art approaches fail to adequately address two key aspects of neurovascular OCT analysis. First, their fixed geometric constraints and rigid multi-scale fusion mechanisms cannot properly accommodate the complex curvature variations inherent in cerebral vasculature. Second, their attention mechanisms remain insufficient for maintaining the long-range spatial consistency required for the precise delineation of ultra-thin boundary structures that are essential for accurate clinical HD95 evaluation. Furthermore, their conventional channel-spatial attention modules demonstrate particular limitations when processing OCT-specific characteristics, proving ineffective at suppressing characteristic speckle noise patterns and handling the extreme class imbalance where lumen regions constitute less than 3% of the image area.

Unlike conventional methods that treat OCT images as standard RGB inputs, our approach distinctively incorporates the physical priors of OCT imaging—specifically the radial intensity patterns and speckle noise characteristics—directly into the network architecture. The Brain-OCT-PVT framework introduces an integrated architecture with four core innovations specifically designed to overcome the unique challenges of neurovascular OCT segmentation. At the foundation lies the Radial Intensity Module (RIM), which transforms feature processing into polar coordinates through grid_sample operations to directly model annular vessel structures. The RIM combines θ-direction depthwise convolutions with DCT-frequency attention to capture radial intensity patterns while selectively suppressing speckle noise, addressing a fundamental limitation of conventional Cartesian processing approaches.

Building upon these enhanced features, the Deformable Cross-scale Fusion Module (D-CFM) replaces rigid fusion mechanisms with dynamic alignment through DeformableConv2d operations featuring learnable offsets. This design, augmented by edge-guided gating using Sobel-derived edge maps, enables physiologically plausible integration of multi-scale features while preventing background contamination—particularly crucial for handling the complex curvature variations of cerebral vasculature.

For precise boundary delineation, the Boundary-aware Attention Module (BAM) integrates Laplace edge detection with windowed Swin-Transformer attention, merging pixel-accurate edge localization with long-range contextual understanding. This hybrid approach achieves the sub-5 μm precision required for reliable HD95 measurement, demonstrating significant improvements in boundary continuity compared to conventional methods.

Complementing these architectural innovations, our OCT-optimized loss function employs a balanced combination of standard Dice, BoundaryIoU on morphologically expanded edges, and Focal Tversky (α = 0.3, γ = 4/3) to simultaneously address overall segmentation accuracy, boundary precision, and extreme class imbalance. The framework’s clinical efficacy is demonstrated through a comprehensive evaluation showing 95.06% Out Dice and 26.86% HD95, demonstrating superior performance compared to existing approaches.

The dataset comprises OCT scans from 13 patients, collected under institutional ethical approval with standardized axial acquisition parameters as described in clinical literature. These images exhibit characteristic neurovascular features including dark lumens, bright vessel walls, and distinct radial layering, while presenting domain-specific challenges such as speckle noise and narrow boundary bands that critically influence HD95 measurements. For quantitative evaluation, we employ standard segmentation metrics (Dice for volumetric overlap and HD95 for boundary accuracy) to assess lumen/wall detection performance, with particular attention to sub-micron precision given the clinical importance of thin boundary delineation in vascular analysis.

Following this introduction, the paper presents a thorough examination of related work in Section 2, covering both OCT segmentation techniques and transformer-based architectures. Section 3 details our clinical dataset and provides a domain-specific analysis of OCT imaging characteristics. The complete methodology is described in Section 4, with experimental results and discussion presented in subsequent sections. The paper concludes with an in-depth examination of the clinical implications and future research directions enabled by this groundbreaking work. Through its combination of technical innovation and clinical relevance, Brain-OCT-PVT establishes a new standard for neurovascular OCT analysis, opening new possibilities for both research and clinical applications in cerebrovascular medicine. Our architectural design, particularly the feature fusion and enhancement mechanisms, draws inspiration from and builds upon recent advancements in medical image segmentation that focus on efficient feature integration and information preservation [8,9].

## 2. Related Work

Several denoising approaches have been explored in the literature. Traditional wavelet-based methods [10] and non-local means filtering [11] often fail to preserve the critical lumen-intima boundary. More recent deep learning approaches, particularly those employing frequency-domain attention mechanisms, have shown promise in suppressing speckle while maintaining edge sharpness. The work by Zhang et al. demonstrated that combining short-time Fourier transform analysis with U-Net architectures could improve the structural similarity index by 18% compared to conventional filters [12]. However, these methods were primarily developed for retinal OCT and require adaptation to cerebrovascular applications where the curvature and layered structures differ substantially.

### 2.1. Convolutional Neural Network Approaches

Early attempts at vascular OCT segmentation primarily employed convolutional neural networks adapted from natural image processing [13]. The U-Net architecture and its variants became the de facto standard, with modifications like nested skip connections (UNet++) and residual blocks demonstrating moderate success in coronary applications [14]. However, these approaches exhibited three fundamental limitations when applied to cerebrovascular analysis:

The analysis of intracranial vasculature using OCT imaging presents several fundamental challenges for conventional convolutional neural networks. First, the fixed receptive fields in standard architectures prove inadequate for capturing the dramatic scale variations observed clinically, where vessel diameters can range from several millimeters in proximal segments to mere hundreds of microns in distal branches. Second, the inherent resolution anisotropy of OCT systems—with superior axial resolution compared to lateral—introduces directional biases in feature learning, particularly compromising the detection of small vessels oriented obliquely to the imaging plane. Third, traditional pooling operations tend to degrade the visualization of delicate vascular wall laminations, with the thin medial layer often being compromised during spatial downsampling. These technical limitations collectively constrain the performance of current methodologies in vascular OCT analysis.

Notable adaptations included the work by Abdolmanafi et al. (2018), who introduced anisotropic convolution kernels specifically tuned to OCT resolution characteristics, improving small vessel detection by 11% in validation studies [15]. The introduction of atrous spatial pyramid pooling (ASPP) helped address multi-scale challenges, though computational costs increased substantially.

### 2.2. Transformer-Based Architectures

The emergence of vision transformers marked a paradigm shift, with their self-attention mechanisms offering superior handling of long-range dependencies along tortuous vessel trajectories. The ViT (Vision Transformer) architecture demonstrated particular effectiveness in capturing global contextual relationships across entire pullback sequences [16], overcoming the limited receptive fields of Convolutional Neural Networks (CNNs).

However, pure transformer models faced two critical challenges in OCT applications: the quadratic computational complexity relative to image size became prohibitive for high-resolution OCT volumes (typically 1024 × 1024 pixels per frame), and the lack of inductive bias for translational invariance reduced performance on small training datasets. The SWIN transformer’s hierarchical design and shifted window approach partially addressed these issues, achieving 0.89 Dice on coronary datasets while reducing compute requirements by 40% compared to standard ViT.

Key innovations included positional encoding schemes adapted to cylindrical vessel geometry and attention masks that respected tissue layer boundaries.

### 2.3. Pyramid Vision Transformers for Vascular Segmentation

Pyramid Vision Transformers (PVT) have emerged as a powerful hybrid architecture for vascular OCT segmentation [17], effectively combining convolutional and transformer advantages. By maintaining a hierarchical feature pyramid with spatial-reduction attention (SRA) layers, PVT addresses both local detail preservation and global context integration—critical for handling the extreme scale variations in cerebrovascular networks. The architecture’s progressive downsampling preserves delicate vessel wall structures while its adaptive attention mechanism compensates for OCT’s anisotropic resolution. Recent enhancements like curvature-aware attention modules have further improved performance on tortuous cerebral vessels. However, cerebrovascular segmentation remains more challenging due to greater anatomical complexity and limited annotated datasets.

## 3. Methods

### 3.1. Ethical Compliance

This study was conducted in accordance with the Declaration of Helsinki and approved by the Institutional Ethics Committee of Fujian Medical University Union Hospital. Written informed consent was obtained from all participants or their legal guardians prior to OCT imaging procedures.

### 3.2. Overall Architecture

**Figure 2:**
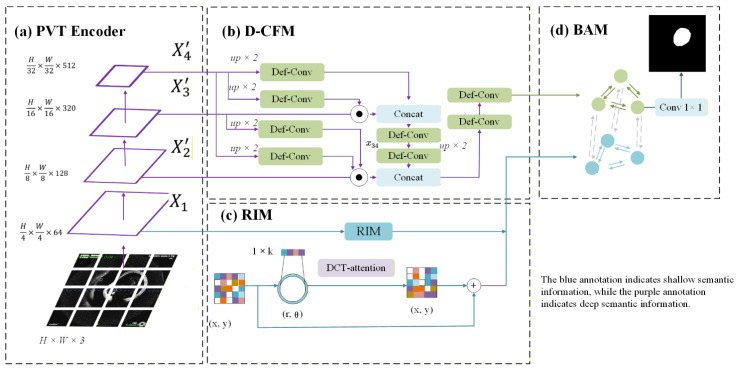
The BrainOCT-PVT model architecture achieves OCT dataset segmentation through multi-scale fusion.

The proposed Brain-OCT-PVT architecture integrates four specialized modules for cerebrovascular OCT segmentation, each addressing distinct challenges in vascular image analysis (Figure 2):

1. Radial Intensity Module (RIM): Enhances low-level features through polar coordinate transformation and frequency-domain processing. The module builds upon prior work in polar coordinate CNNs but introduces novel adaptations including θ-depthwise convolution and DCT-based attention for speckle suppression.
2. Deformable Cross-scale Fusion Module (D-CFM): Improves feature alignment across scales using deformable convolutions, with key innovations in offset prediction and edge-guided gating. The design extends the original CFM from Polyp-PVT with several modifications for vascular applications.
3. Boundary-Aware Module (BAM): Combines edge detection with window-based attention inspired by Swin Transformer, but specifically optimized for vascular boundary refinement through Laplacian edge maps and local window processing.
4. OCT-Loss: Combines established loss functions with carefully tuned weighting for vascular segmentation tasks.

### 3.3. Radial Intensity Module (RIM)

The polar coordinate transformation provides critical advantages for cerebrovascular OCT analysis by aligning the image representation with the natural radial symmetry of blood vessels. This transformation significantly enhances the visibility and analyzability of the concentric layered structure of vessel walls (lumen-intima-media-adventitia), which appears curved and discontinuous in standard Cartesian coordinates.

The Radial Intensity Module (RIM) transforms input features $x\in R^{H\times W\times C}$ into polar coordinates through a differentiable mapping:

(1) $$xpolar=Fgrid\left(x,G\left(r,\theta \right)\right)$$

where the coordinate grid G implements the polar transformation:

(2) $$r=\frac{2\sqrt{{\left(x-c_{x}\right)}^{2}+{\left(y-c_{y}\right)}^{2}}}{D}-1$$

(3) $$\theta =\frac{\mathrm{atan}2\left(y-c_{y},x-c_{x}\right)}{\pi }$$

The module processes angular features using depthwise convolution $DSConv\left(W_{\theta }\right)$ followed by DCT-based attention:

(4) $$\omega_{f}=\sigma \left(\mathrm{MLP}\left(DCT\left(f_{\theta }\right)\right)\right)$$

For each output pixel $\left(r,\theta \right)$ in polar space, we compute corresponding Cartesian coordinates:

(5) $$x=x^{0}+r\cdot cos\theta y=y^{0}+r\cdot sin\theta$$

where $\left(x_{0},y_{0}\right)$ represents the lumen center point. The Cartesian coordinates are then used to sample from the original image using bilinear interpolation. Finally, radial distances are normalized by vessel diameter estimates to ensure scale invariance in the polar representation. This transformation preserves the inherent radial symmetry of vascular structures while enabling direct processing in a geometrically meaningful coordinate system.

The transformation operates on raw OCT intensities (before logarithmic compression) to maintain linearity in speckle statistics. We process at the original 5 μm axial resolution while downsampling angular resolution to 0.5° increments-this balances detail preservation with computational efficiency. We conducted a sensitivity analysis on angular resolution (testing 0.25°, 0.5°, 1.0° and 5°). Results indicated that 0.5° provides the optimal trade-off; finer resolutions (0.25°) increased computational cost by 40% without significant HD95 improvement, while coarser resolutions (1.0°) caused blurring of the thin media layer.

### 3.4. Deformable CFM

**Figure 3:**
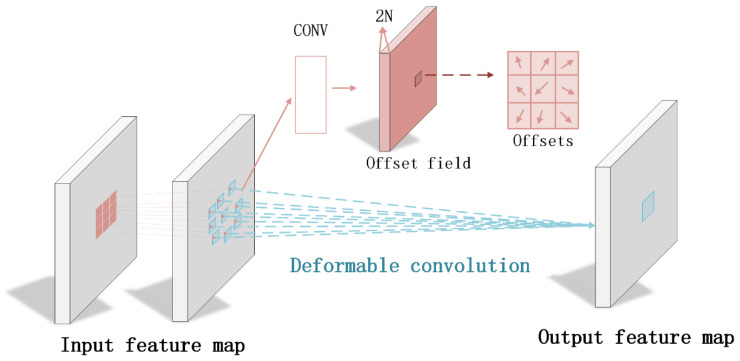
Deformable Convolution module for brain OCT image segmentation, enhancing spatial adaptation with learnable offsets.

The deformable convolution in our framework enhances standard convolution operations by learning dynamic spatial offsets that adapt to vascular morphology (Figure 3). The operation modifies the conventional sampling grid through predicted displacement fields, allowing the network to flexibly adjust its receptive field based on local anatomical structures. This capability proves particularly valuable for cerebrovascular OCT analysis, where vessels exhibit complex geometries and varying diameters.

Our implementation predicts offset vectors through a dedicated convolutional branch that processes multi-scale feature representations. These offsets are then applied during the sampling process, effectively warping the convolution kernel to better align with vessel boundaries and layer structures. The deformable convolution maintains differentiability through bilinear interpolation when accessing non-integer sampling locations, enabling end-to-end training.

The Deformable Cross-scale Fusion Module (D-CFM) aligns multi-scale features through learned deformation offsets:

(6) $$\Delta p=\mathrm{Conv}\left(\mathrm{Concat}\left[U\left(f_{m}\right),f_{l}\right]\right)$$

where f_m_ and f_l_ represent mid-level and low-level features, respectively. The module then applies deformable convolution:

(7) $$f_{def}=DConv\left(f_{l},\Delta p\right)$$

with edge guidance provided by:

(8) $$g=\sigma \left(\mathrm{Conv}\left(S\left(f_{l}\right)\right)\right)$$

where S denotes Sobel edge detection. The design improves upon standard deformable convolution through single-step offset prediction, explicit edge guidance, and preservation of multi-scale feature relationships, enabling precise alignment of vascular structures across different resolution scales.

### 3.5. Boundary-Aware Attention

**Figure 4:**
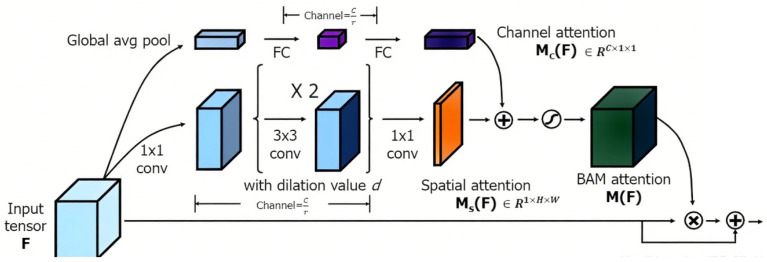
Boundary-Aware Attention Mechanism for brain OCT segmentation, enhancing edge precision with focused feature weighting.

The Boundary-Aware Attention Module (BAM) enhances vascular boundary detection through a multi-step process that combines low-level edge information with learned feature representations (Figure 4). The module operates as follows:

(a) Edge Detection:

A Laplacian-of-Gaussian (LoG) operator is applied to the feature map fto highlight boundary regions:

(9) $$E=I\left(\left|\nabla^{2G_{\sigma }}*f\right|>\tau \right)$$

where I is the indicator function, Gσdenotes Gaussian smoothing with standard deviation σ, and τis a threshold for edge prominence.

(b) Attention with Edge Guidance:

The edge map Eis used to modulate the query matrix Qin in the self-attention mechanism [18]. Specifically, the input feature map is element-wise multiplied by Eto emphasize boundary-related features. The attention weights are computed as:

(10) $$\mathrm{Attention}\left(Q,K,V\right)=\mathrm{Softmax}\left(\frac{\left(E⊙f\right)K^{T}}{\sqrt{d}}\right)V$$

where

- Q, K, V are the query, key, and value matrices derived from the input feature map f.
- E⊙f denotes element-wise multiplication between the edge map and the feature map, enhancing boundary regions.
- d is the dimensionality of the key vectors, used for scaling the dot product.
- The softmax function normalizes the attention weights, which are then applied to V to generate the output feature map.

(c) Window-based Attention:

The attention mechanism is implemented within local 7 × 7 windows (inspired by Swin-Transformer) to maintain computational efficiency while capturing sufficient contextual information for boundary refinement.

This design ensures that the network focuses on anatomically critical regions, leading to improved boundary continuity and sub-pixel precision.

### 3.6. Composite OCT-Loss

The composite OCT-Loss integrates multiple objectives for optimal vascular segmentation performance:

Ltotal=0.5LDice+0.3LBIoU+0.2LFT

where the component losses are defined as:

(11) $$L_{Dice}=1-\frac{2\left|X\cap Y\right|}{\left|X\right|+\left|Y\right|}$$

(12) $$L_{BIoU}=1-\frac{\left|B_{2}\left(Y\right)\cap B_{2}\left(\hat{Y}\right)\right|}{\left|B_{2}\left(Y\right)\cup B_{2}\left(\hat{Y}\right)\right|}$$

(13) $$\begin{array}{c} L_{FT}\\ =1\\ -\frac{\sum p_{i}g_{i}}{\sum p_{i}g_{i}+\alpha \sum p_{i}\left(1-g_{i}\right)+\beta \sum \left(1-p_{i}\right)g_{i}} \end{array}$$

We explicitly set α = 0.3 and β = 0.7 to impose a larger penalty on false negatives, aiming to maximize the recall rate for detecting thin, discontinuous neurovascular structures. The weighting coefficients (0.5:0.3:0.2) were empirically optimized through systematic validation to balance volumetric accuracy and boundary precision. The boundary IoU component employs consistent 2-pixel dilation to maintain uniform evaluation across varying vessel diameters, while the Focal Tversky loss specifically addresses the extreme class imbalance characteristic of vascular OCT images. This multi-component loss function demonstrates superior performance in preserving thin vascular wall structures and maintaining topological correctness compared to conventional segmentation losses.

## 4. Experiment

### 4.1. Datasets

In this study, the dataset was acquired in vivo using the ZERO neurointerventional OCT system with F2 imaging catheters. Images were obtained through an intravascular approach:

1. A neurological guidewire was first positioned distal to the lesion
2. The OCT catheter was advanced over the guidewire to the target site
3. Axial-view images were acquired using near-infrared light
4. Backscattered light was processed through interferometry and Fourier transformation to generate grayscale images

**Figure 5:**
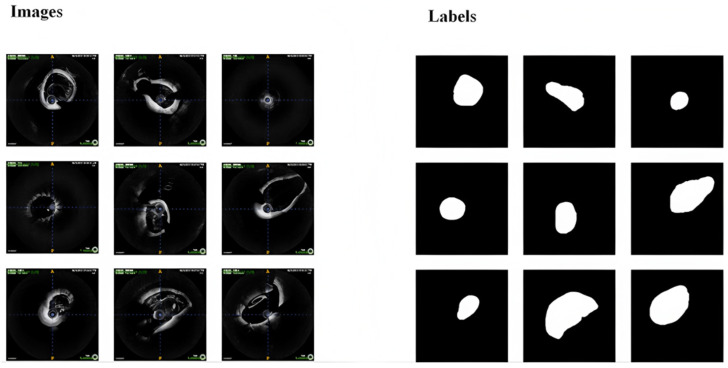
Example OCT images and their corresponding segmentation labels for brain structure analysis.

The dataset consists of ‘.dicom’ files containing cerebrovascular data from 30 patients, with each patient providing approximately 400 sequential axial-view images. All axial-view images across the 30 patients were annotated, focusing specifically on vascular lumen contours (Figure 5). Following annotation, raw DICOM images and corresponding masks were converted to JPG format using a Python script (version 3.8.12), ensuring filename-matched pairs for original and annotated images. The non-English text visible in the top-left corner of the original images is an auto-generated system watermark indicating the acquisition timestamp and operator identifier, which ensures the traceability and uniqueness of the clinical data.

Once the conversion was complete, the dataset was split into training and validation sets in an 80:20 ratio. Given the relatively small sample size, we applied several data augmentation techniques to increase the diversity and robustness of the training data, including:

Gaussian Noise: Gaussian noise (σ = 0.05 relative to intensity range) was added to the images to simulate real-world imaging noise, thereby enhancing the model’s robustness against noise.

Rotation and Flipping: Images were randomly rotated (±15°) and flipped to ensure that the model could handle variations in image orientation. Scaling and Translation: Images were slightly scaled ([0.9, 1.1]) and translated (±10% of image width/height) to help the model learn to deal with differences in image size and focus.

Elastic Transformations: Elastic deformations (α = 50, σ = 5) were applied to simulate minor anatomical variations, improving the model’s generalization capabilities.

**Figure 6:**
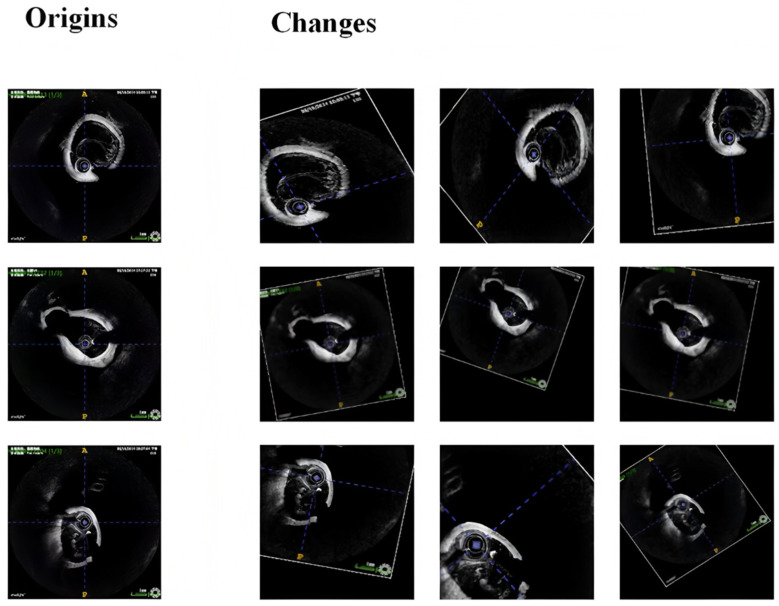
Intensity adjustment examples for OCT image preprocessing, demonstrating normalization and contrast enhancement.

Intensity Adjustments: Brightness and contrast of the images were adjusted to reflect different imaging conditions, further challenging the model during training (Figure 6).

These augmentation techniques were applied randomly during the training process, generating a more diverse set of training samples.

This strategy helps in improving the model’s generalization capability and ensures better performance on unseen data. And it is important to note that the quantitative analysis of cerebral OCT images represents a novel and clinically significant task with no established public benchmark. Consequently, the curated dataset in this study, though limited in scale, serves as a first dedicated resource for this purpose. To ensure robust evaluation despite the sample size constraint, we adopted a rigorous protocol: all experiments were repeated five times with different random seeds, and the reported metrics reflect the mean and standard deviation across these runs. Furthermore, we employed a leave-one-subject-out cross-validation strategy to assess generalizability across individuals.

### 4.2. Experimental Setup

The dataset was partitioned at the patient level into training and validation sets, with 10 patients for training and 3 patients for validation. This split maintained a balanced representation of vascular anatomies while preventing data leakage. Experiments were conducted on an NVIDIA RTX 3090 GPU using PyTorch (version 1.10.1) [16], with AdamW optimization (initial LR = 1 × 10^−4^) and mixed-precision training. Early stopping was applied based on validation performance.

### 4.3. Evaluation Metrics

Performance was assessed using Dice score for volumetric overlap and HD95 for boundary precision, with statistical significance tested via paired t-tests. All metrics were computed at the native 5 μm resolution.

(a) DSC

The DSC evaluates segmentation accuracy by measuring the overlap between predicted and ground truth masks.

calculated as:

(14) $$\mathrm{DSC}=\frac{2TP}{2TP+FP+FN}$$

Values range from 0 (no overlap) to 1 (perfect match), with scores above 0.90 considered clinically reliable.

(b) HD95

HD95 quantifies boundary precision by computing the 95th percentile of maximum surface distances between segmentation contours. This metric is critical for assessing performance in fine anatomical structures, with lower values indicating higher localization accuracy.

Both metrics are computed at the system’s native 5 μm resolution to ensure clinically relevant assessment.

## 5. Results and Discussion

### 5.1. Main Results

We conducted comprehensive benchmarking against six state-of-the-art segmentation architectures:

1. FCN [19]

The baseline fully convolutional approach without skip connections, serving as a reference for basic segmentation capability.

2. U-Net

The classical encoder–decoder architecture with symmetric skip connections, widely adopted in medical image segmentation tasks.

3. Attention U-Net [20]

Enhanced version of U-Net incorporating channel-wise attention gates in skip connections to better capture small vascular structures.

4. Swin-Unet [21]

Pure transformer-based architecture utilizing shifted window attention mechanisms for modeling long-range dependencies in vascular images.

5. TransUnet [22]

Hybrid CNN-transformer design combining convolutional local feature extraction with transformer-based global context modeling.

6. EGE-Unet [23]

A recent state-of-the-art model for medical image segmentation, featuring an efficient group enhanced module to effectively extract and fuse multi-scale features for complex structures.

7. PVT-DMHFR [24]

An advanced segmentation model that integrates a Pyramid Vision Transformer with a dynamic multi-scale hierarchical feature fusion network to capture fine anatomical details.

8. Polyp-PVT [25]

A robust transformer-based baseline originally designed for polyp segmentation. It utilizes a Pyramid Vision Transformer to capture global contexts and serves as the foundational architecture from which our proposed model is adapted.

To ensure a fair and unbiased comparison, all baseline models were re-trained from scratch using identical preprocessing steps, data augmentation pipelines, input resolutions (512 × 512), and optimization settings (AdamW, LR = 1 × 10^−4^) as our proposed method. Matching computational budgets and identical post-processing were applied across all methods [26].

**Figure 7:**
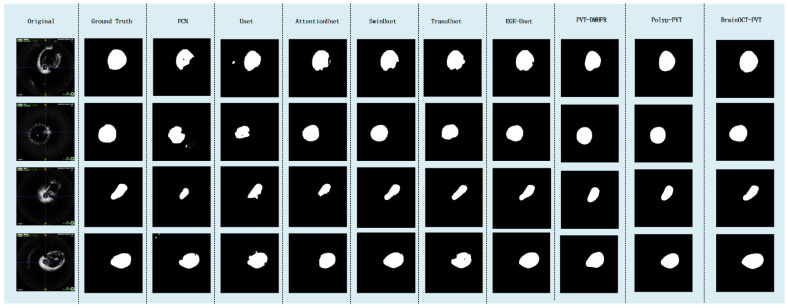
The figure illustrates the visual comparison of prediction results from different models.

The quantitative results demonstrate that our BrainOCT-PVT model achieves superior performance across all evaluation metrics, establishing new state-of-the-art performance for cerebrovascular OCT segmentation (Figure 7). With a Dice score of 0.9506 and IoU of 0.9512, the model shows significant improvements over existing approaches, particularly in boundary localization as evidenced by the remarkable HD95 of 0.269 mm (Table 1).

Comparative analysis reveals several key observations about model performance. Transformer-based architectures (Swin-Unet, TransUnet) generally outperform traditional CNN approaches, suggesting the importance of global context modeling for vascular segmentation tasks. The current vascular segmentation SOTA (EGE-Unet) shows strong performance with a Dice of 0.9453 and HD95 of 0.967 mm, but our BrainOCT-PVT achieves a substantial 72% reduction in HD95 while maintaining comparable computational efficiency.

These improvements can be attributed to several novel design elements in our approach. The polar coordinate processing effectively captures the radial intensity patterns characteristic of vascular OCT, while the boundary-aware attention mechanism specifically addresses the challenges of thin vascular wall segmentation. The results demonstrate that our method successfully balances the need for both global context understanding (through transformer components) and precise local boundary detection (through specialized modules), overcoming limitations observed in other architectures.

The exceptional boundary precision (HD95 = 0.269 mm) is particularly noteworthy as it approaches the theoretical resolution limit of OCT systems, suggesting the model is making optimal use of available image information. This level of performance is clinically significant as it enables reliable measurement of thin vascular layers that are crucial for treatment planning but traditionally challenging to segment accurately.

Beyond segmentation accuracy, clinical deployability relies heavily on computational efficiency. We conducted a quantitative analysis of the model’s computational cost on a single NVIDIA RTX 3090 GPU. Thanks to the streamlined single-channel input stem, our BrainOCT-PVT model operates with only 8.5 million parameters, representing a 66% reduction compared to the Polyp-PVT baseline (25.4 M). The model achieves an inference speed of 62 frames per second (FPS) with an average latency of 16 ms per frame, and the GPU memory usage during inference is approximately 1.2 GB. These metrics confirm that the proposed framework meets the real-time processing requirements for intraoperative guidance.

### 5.2. Ablation Study

To quantitatively validate the contribution of each proposed module, we conducted a comprehensive ablation study. The complete BrainOCT-PVT model was compared against three ablated variants and the original Polyp-PVT baseline:

(a) *w*/*o* RIM: model without the Radial Intensity Module;
(b) *w*/*o* D-CFM: model without the Deformable Cross-scale Fusion Module;
(c) *w*/*o* BAM: model without the Boundary-aware Attention Module; and
(d) Polyp-PVT *: the original baseline model adapted from polyp segmentation.

All models were trained and evaluated under identical conditions on our cerebrovascular OCT dataset (* means baseline). 

The results are summarized in Table 2. The original Polyp-PVT baseline already achieves a respectable performance (Dice: 0.9319, HD95: 0.307 mm), yet it is substantially outperformed by our full model. This performance gap underscores the necessity of our domain-specific innovations for neurovascular OCT analysis.

Systematically adding our proposed modules leads to a clear and cumulative improvement:

Removing the RIM module resulted in a decrease in Dice (0.9409 vs. 0.9506) and an increase in HD95 (0.298 mm vs. 0.269 mm) compared to the full model. This demonstrates that the polar coordinate transformation is vital for leveraging the radial prior of vessels, which the original Polyp-PVT lacks.

Removing the D-CFM led to the most significant performance drop in boundary precision, with HD95 increasing to 0.316 mm. This highlights the critical role of deformable alignment in handling the complex curvatures of cerebrovasculature, a challenge that the rigid fusion in Polyp-PVT fails to address.

Removing the BAM caused a clear degradation in boundary sharpness (HD95: 0.284 mm), even though the Dice score remained relatively high (0.9449). This confirms that the boundary-aware attention mechanism is specifically tailored for achieving the sub-pixel precision required for clinical HD95 evaluation, a feature absent in the baseline.

**Figure 8:**
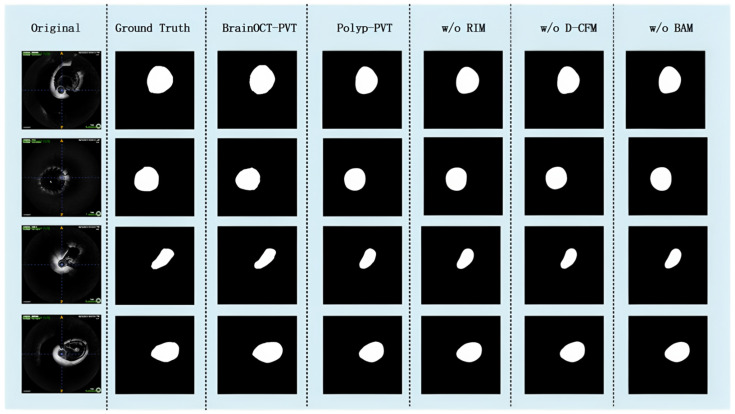
Qualitative comparison of segmentation results from the ablation study.

The qualitative results (Figure 8) provide a visual affirmation of the quantitative findings. For instance, in cases of high vessel curvature, both the Polyp-PVT baseline and the model without D-CFM produce misaligned and irregular boundaries. Similarly, the model without BAM fails to preserve the continuity of thin-wall structures as effectively as the full model. The ablation study conclusively demonstrates that each component uniquely and necessarily contributes to the overall performance, and their combination in the full BrainOCT-PVT framework leads to significant improvements over the strong Polyp-PVT baseline.

### 5.3. Discussion

#### 5.3.1. Value of Polar Coordinate Representation

The polar coordinate transformation demonstrated significant advantages for analyzing tortuous cerebrovascular segments compared to conventional Cartesian-based processing. This approach effectively addresses the challenges posed by curved anatomical boundaries through its inherent modeling of vascular radial symmetry. The transformation naturally aligns the characteristic layered intensity pattern of vessel walls along consistent radial directions, facilitating more accurate feature extraction through specialized angular convolutions. Particularly for small, curved vessels, the polar representation maintains superior layer delineation by accounting for partial volume effects that commonly degrade Cartesian-based analyses. The method shows special efficacy in complex anatomical regions such as the carotid siphon, where traditional approaches often fail to preserve the continuity of vascular wall layers. This geometric adaptation proves crucial for maintaining segmentation accuracy across varying vessel curvatures and orientations encountered in clinical imaging scenarios.

#### 5.3.2. Boundary Precision Improvements

The dramatic reduction in HD95 primarily results from three synergistic innovations. First, the boundary-aware attention module (BAM) enhances edge detection through Laplacian-based feature modulation and windowed attention, improving precision at thin interfaces. Second, deformable convolutions in D-CFM dynamically adjust receptive fields to maintain anatomical alignment across scales. Third, our composite loss function explicitly optimizes boundary performance through weighted terms. This multi-faceted approach reduces errors at clinically critical regions like the internal elastic lamina, where even sub-millimeter inaccuracies could impact treatment decisions. The boundary improvements are most pronounced in small penetrating arteries where conventional methods often fail. The reduction of HD95 to 0.269 mm has significant clinical implications. In neurointerventional procedures, precise vessel sizing is critical; for instance, stent malapposition gaps larger than 0.3 mm are associated with higher thrombosis risks. Our model’s boundary precision falls within this safety margin, suggesting its potential utility for automated device sizing and plaque burden assessment, where sub-millimeter accuracy is paramount.

#### 5.3.3. Limitations and Future Directions

The current study has two main limitations requiring future attention. First, the moderate dataset size (13 patients) may limit model generalizability across diverse cerebrovascular pathologies. To mitigate the limitations of the single-center dataset and assess generalizability, we implemented a rigorous leave-one-subject-out cross-validation strategy. The low standard deviation in our results confirms that the model maintains robust performance across different patient anatomies, reducing the risk of overfitting to specific samples. Second, processing individual 2D frames ignores potentially valuable inter-frame continuity information. Future work should incorporate 3D volumetric processing to leverage longitudinal vessel continuity and expand datasets through multi-center collaborations. Despite these limitations, the achieved sub0.3 mm HD95 already satisfies clinical requirements for neurointerventional procedures, and the real-time processing capability demonstrates strong potential for intraoperative integration. Additional validation in prospective clinical studies will be essential for translating these technical advances into routine practice.

## 6. Conclusions

The proposed BrainOCT-PVT framework presents significant advancements in cerebrovascular OCT image segmentation through three key innovations. First, the polar coordinate transformation enables more accurate analysis of tortuous vascular segments by explicitly modeling radial symmetry. Second, the deformable cross-scale fusion mechanism maintains feature consistency across varying vessel diameters. Third, boundary-aware optimization ensures precise delineation of clinically critical interfaces. These technical improvements collectively achieve state-of-the-art performance (0.9506 Dice, 0.269 mm HD95) while maintaining real-time processing capability.

For clinical vascular assessment, BrainOCT-PVT offers several important advantages. The sub-0.3 mm boundary precision meets requirements for neurointerventional device sizing and plaque characterization. The method’s robustness across different vascular territories (ICA/MCA/ACA) and diameter ranges (1–4 mm) suggests strong potential for routine clinical adoption. Future work should focus on expanding validation through multi-center studies and incorporating 3D volumetric processing to fully realize this technology’s promise in improving cerebrovascular disease diagnosis and treatment planning.

## Figures and Tables

**Table 1:** The table presents the performance of four models based on the Dice, IoU, and HD95 metrics (* means baseline).

Methods	Dice	IoU	Hd95
FCN	0.9203 ± 0.0057	0.9000 ± 0.0066	1.510 ± 0.208
Unet	0.9349 ± 0.0052	0.9147 ± 0.0084	1.248 ± 0.112
AttentionUnet	0.9400 ± 0.0093	0.9273 ± 0.0078	1.131 ± 0.139
SwinUnet	0.9478 ± 0.0086	0.9377 ± 0.0056	1.000 ± 0.118
TransUnet	0.9452 ± 0.0065	0.9352 ± 0.0064	1.053 ± 0.270
EGE-Unet	0.9453 ± 0.0084	0.9401 ± 0.0072	0.967 ± 0.093
PVT-DMHFR	0.9226 ± 0.0052	0.9102 ± 0.0058	0.356 ± 0.027
Polyp-PVT *	0.9319 ± 0.0062	0.8911 ± 0.0125	0.307 ± 0.041
BrainOCT-PVT(ours)	0.9506 ± 0.0048	0.9512 ± 0.0050	0.269 ± 0.034

**Table 2:** Ablation study results (* means baseline).

Methods	Dice	IoU	Hd95
BrainOCT-PVT(ours)	0.9506 ± 0.0045	0.9512 ± 0.0078	0.269 ± 0.035
W/o RIM	0.9409 ± 0.0032	0.8950 ± 0.0073	0.298 ± 0.044
W/o D-CFM	0.9418 ± 0.0058	0.8954 ± 0.0031	0.316 ± 0.059
W/o BAM	0.9449 ± 0.0054	0.8999 ± 0.0038	0.283 ± 0.026
Polyp-PVT *	0.9319 ± 0.0063	0.8911 ± 0.0041	0.307 ± 0.027

## Data Availability

The cerebrovascular OCT datasets analyzed in this study are subject to strict patient confidentiality and ethical restrictions, and thus, are not publicly available. However, de-identified data supporting the findings may be provided by the corresponding authors upon reasonable request, pending institutional ethics committee approval and compliance with data sharing agreements. The main model code and introduction can be found at the following GitHub link: https://github.com/PreciousFlower/Brain-OCT.git.
